# Omalizumab prevents respiratory illnesses in non‐atopic chronic spontaneous urticaria patients: A prospective, parallel‐group, pilot pragmatic trial

**DOI:** 10.1002/clt2.12279

**Published:** 2023-07-13

**Authors:** George N. Konstantinou, Indrashis Podder, Dimitrios Karapiperis

**Affiliations:** ^1^ Department of Allergy and Clinical Immunology 424 General Military Training Hospital Thessaloniki Greece; ^2^ Department of Dermatology College of Medicine and Sagore Dutta Hospital Kolkata West Bengal India; ^3^ Department of Infectious Diseases 424 General Military Training Hospital Thessaloniki Greece

**Keywords:** atopy, chronic spontaneous urticaria, interferon, non‐atopic, omalizumab, respiratory symptoms, viral

## Abstract

**Background:**

Omalizumab is the recommended treatment for antihistamine‐refractory chronic spontaneous urticaria (CSU) and severe allergic asthma. In addition, it has been shown to reduce the frequency of viral respiratory infections in allergic asthma. Respiratory illness is a known trigger for asthma and CSU.

**Objectives:**

To explore whether the antiviral effect of omalizumab may be extended to CSU patients independent of their atopic status.

**Methods:**

We conducted a prospective parallel‐group pilot pragmatic trial including 30 non‐allergic and non‐atopic CSU patients (cases) under omalizumab 300 mg Q4‐weeks (due to refractory to H1‐antihistamines) and 30 age‐matched healthy controls. All CSU patients had to have a weekly urticaria activity score UAS7 <15 at least 4 weeks before recruitment. Using the self‐filled validated Jackson scale, we evaluated all study participants for common cold symptoms. All cases and controls rated weekly their respiratory symptoms. An increase in the symptom score of at least 4 points compared to baseline (defined as the minimum weekly report of symptoms) was considered an episode suggestive of a viral infection of the upper respiratory tract (URT). The patients were follow‐up every 4 weeks throughout the study period (10 months).

**Results:**

CSU patients under omalizumab reported fewer episodes suggestive of an URT viral infection than the healthy controls (median of reported episodes: 0 vs. 1, inter‐quartile range 0–1 vs. 1–1, min–max: 0–3 vs. 0–4, respectively; *p* = 0.0095). The duration of each episode was the same in both cases and controls.

**Conclusions:**

Omalizumab can reduce the number of common cold episodes in CSU patients and consequently may minimize viral‐related CSU exacerbations. This beneficial effect is exerted independently of the atopic status, even in non‐asthmatic individuals or non‐allergic patients without any evidence of respiratory susceptibility. Further large‐scale studies are needed to validate the current findings and elucidate the underlying relevant pathophysiology.

## 1 INTRODUCTION

Urticaria is a skin disorder characterized by the sudden and unpredictable appearance of pruritic wheals, with or without angioedema, usually resolving within 24 h. It is considered chronic when symptoms persist for more than 6 consecutive weeks.^1^ In almost 70%–90% of patients, no specific trigger can be identified, such as a physical stimulus, food, allergen, or drug, and these cases represent chronic spontaneous urticaria (CSU).^2^, ^3^

The current guidelines recommend standard doses of second‐generation (non‐sedative) H1 antihistamines (sg‐AH) as the first‐line treatment and their incremental up‐dosing up to four‐fold in case of inadequate response in 2–4 weeks.^1^ However, almost 60% of patients remain uncontrolled with the standard antihistamine dose, and most of them do not respond even with up‐dosed sg‐AHs.^4^ In such patients, omalizumab (300 mg Q4‐weeks) is the approved second‐line treatment with up‐dosing up to 600 mg Q4‐weeks in refractory patients.^1^

Omalizumab is an anti‐IgE humanized monoclonal antibody, which binds free IgE and downregulates high‐affinity FcεR1 receptors on effector mast cells and basophils, preventing degranulation. As these cells are the cornerstone in the pathogenesis of various allergic diseases, including CSU and asthma, omalizumab clinically improves CSU by blocking their downstream effects.^5^ In addition to CSU and severe asthma, omalizumab is also approved for the treatment of nasal polyposis and, in a few countries, for allergic rhinitis.^6^

Patients with asthma are more prone to respiratory viral infections and virus‐induced disease exacerbation, particularly in those with high serum IgE and severe disease.^7^, ^8^ The possible mechanism involves impaired type I interferon (IFN‐α/β) and interferon III (IFN‐λ) response of epithelial and plasmacytoid dendritic cells (pDCs) in atopic patients, which comprise an essential component of innate antiviral immunity.^9^ Additionally, in atopic/allergic individuals, the expression of the high‐affinity IgE receptor FcεR1 and the Toll‐like receptor 7 (TLR7 – an essential receptor for detecting viruses and mounting antiviral innate immune response) show an inversely proportional relationship, thus making these patients more prone to viral infections.^10^ Furthermore, asthma patients show Th2 skewing of their adaptive immunity, and respiratory viruses possibly act synergistically with Th2 immunity to cause infection.^11^

Viral infections have been associated with acute urticaria onset.^12^ Moreover, HHV‐6 infection has been associated with CSU,^13^ and COVID‐19 with CSU exacerbations with higher rates in patients with severe COVID‐19.^14^ However, there are no large‐scale studies to evaluate if CSU patients are more susceptible to microbial infections and if a particular underlying pathomechanism can suggest a causal relationship between a viral infection and a CSU exacerbation.

Recently, researchers have highlighted the exciting antiviral effect of omalizumab as it ameliorates the inadequate antiviral response in allergic patients by sequestering free IgE and enhancing pDC‐IFN secretion by reducing FcεR1 receptors on mast cells and basophils.^8^ However, this antiviral effect has only been studied in patients with allergic asthma, without evidence of another IgE‐mediated or associated disorder.^15^, ^16^, ^17^

The present study aims to determine whether this antiviral effect may be extrapolated to CSU by evaluating the impact of omalizumab on respiratory infections in non‐allergic, non‐atopic, CSU patients without asthma, rhinitis, or rhinosinusitis.

## 2 MATERIALS AND METHODS

### 2.1 Study design and setting

We conducted a prospective, parallel‐group, pilot pragmatic trial at the departments of Allergy and Clinical Immunology and Infectious Diseases of a tertiary care center in Greece. Requisite approval was obtained from the Institutional review board (424 General Military Training Hospital No:12840/2015), and written informed consent was obtained from all parents. The study was conducted according to Helsinki principles and Strengthening the Reporting of Observational Studies in Epidemiology (STROBE) reporting guidelines for case‐control studies.^18^ The study duration was 10 months (September 2015 to August 2016).

### 2.2 Study participants

**FIGURE 1:**
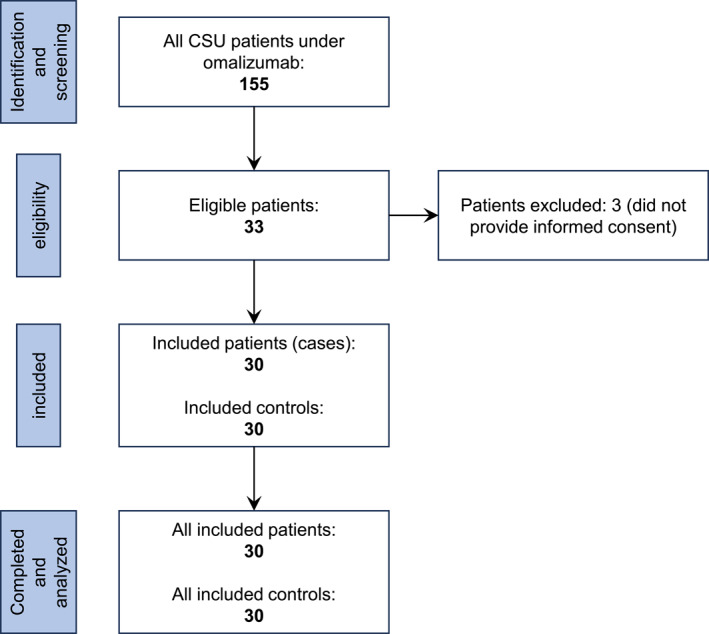
Flow diagram of included and excluded chronic spontaneous urticaria (CSU) patients treated with omalizumab and controls.

We included non‐allergic, non‐atopic adult (>18 years) CSU patients (cases), irrespective of gender, who were under omalizumab because of CSU refractory to regular doses of second‐generation H1 antihistamines (sgAH). Eligible patients should have been urticaria‐free (UAS7 = 0), have well‐controlled urticaria (UAS7 = 1–6), or mild activity CSU (UAS7 = 7–15) at least a month before recruitment. Atopy was defined with either positive skin prick (SPTs) or specific IgE to common environmental and food allergens for the patients before omalizumab was started and for the controls at enrollment in the study (or day 0). Known history of chronic, seasonal, or episodic respiratory illnesses such as asthma (allergic or non‐allergic), allergic rhinitis or chronic rhinitis/rhinosinusitis, any immunodeficiency or systemic disorder, smokers and refusal to provide informed consent comprised our exclusion criteria. We also included a group of age‐matched healthy controls from the household members of patients (spouse, partner, relative, roommate, etc.) who were closest in age and fulfilled the exclusion criteria. If a patient was living on his or her own, they were excluded from the study. Figure 1 depicts the low diagram of the finally included patients and controls.

### 2.3 Assessment of study participants

On day 0, each eligible patient was subjected to a detailed history regarding their demographic details, duration of CSU, and history of atopy. General, dermatological, and systemic examinations were performed to rule out any exclusion criteria. Routine biochemistry and hematology testing were performed for all patients.

The evaluation of the respiratory symptoms in each patient was based on Jackson's cold scale.^19^ Five common symptoms (runny nose, stuffy nose, sore throat, sneezing, and cough) were graded on a scale of 0–3 based on their severity (absent, mild, moderate, or severe, respectively), and a weekly cumulative score was obtained. This scale was filled by patients and their age‐matched controls every 7 days to reflect symptoms over the past week and instructed to bring their filled scales at follow‐up visits (on day 0, the scale was filled by the patient in front of the investigator—baseline measurement). Based on a previous study by Esquivel et al.,^15^ an increase in the symptom score by at least 4 points compared to baseline (defined as the minimum weekly report of symptoms) was considered suggestive of an upper respiratory tract viral infection, as all examined individuals were non‐atopic and had no current or past history of respiratory allergies.

As these patients were refractory to regular doses of sgAHs, we administered omalizumab (300 mg Q4‐weeks, SC) as per current guidelines.^1^ All study participants (cases and controls) were recruited at the beginning of the autumn (September to October) and were followed up on a regular basis every 4‐week at omalizumab administration at the end of the following summer (July to August). At each visit, the filled respiratory scales were checked to evaluate respiratory symptoms over the past 4 weeks. As previously described, a minimum 4‐point increase in the symptom score compared with baseline (defined as the minimum weekly report of symptoms) indicated respiratory illness.^15^

### 2.4 Variables

The following variables were evaluated: age, gender, total serum IgE level, the vaccination rate for influenza virus, and the number of episodes of upper respiratory tract infections (in both cases and controls). The duration of CSU and omalizumab administration were additionally assessed in cases. The primary dependent variable was the number of episodes of URT infections in both cases and controls, the association of which was examined with the omalizumab administration in cases (independent variable).

### 2.5 Bias

The number of URT infections in both cases and controls was assessed using a self‐filled questionnaire by the subjects, so the possibility of recall‐bias cannot be ruled out. Additionally, several environmental confounding factors could be present, such as indoor pollution, passive smoking, and type of occupation or place of work, which were beyond our control.

### 2.6 Statistical analysis

Data were tested for normality using the Kolmogorov‐Smirnov test. Numerical data were represented as mean ± SD or median (inter‐quartile range [IQR]), while categorical data were expressed as proportions or percentages. We used the paired *t*‐test to analyze numerical data (to compare the cases with the matched controls), while the Chi‐square and Fisher's exact tests were used for categorical data. We used the statistical software Stata v 10.0 for analysis and Graph‐pad Prism for drawing the graphs.

## 3 RESULTS

Among 155 Caucasian patients with CSU under omalizumab in our department during that period, 33 were eligible for the study. Three of them were excluded because they did not provide informed consent. We included 30 CSU patients (mean ± SD, 45 ± 16.6 years old; M:F 11:19) and 30 (29 spouses and one partner) healthy controls (mean ± SD, age 46.4 ± 9.6 years; M:F 21:9). Both groups were age‐matched (*p* = 0.332). All cases were active or retired Greek Army Force personnel. The mean ± SD disease duration of CSU was 1.2 ± 0.8 years, and omalizumab administration 0.9 ± 0.5 years. CSU patients had median total serum IgE levels before starting omalizumab of 176.5 IU/ml (range 10–1032, IQR: 65–251 IU/ml), significantly higher than the healthy controls with a median total IgE of 58.5 IU/ml (range 10–110, IQR 49–71 IU/ml, *p*‐value < 0.001). The vaccination rate for influenza viruses was similar between patients and controls (only 3 CSU patients and 2 controls were unvaccinated during the study, these two controls were matched with the two out of the three unvaccinated CSU patients). None of the patients were lost to follow‐up.

**FIGURE 2:**
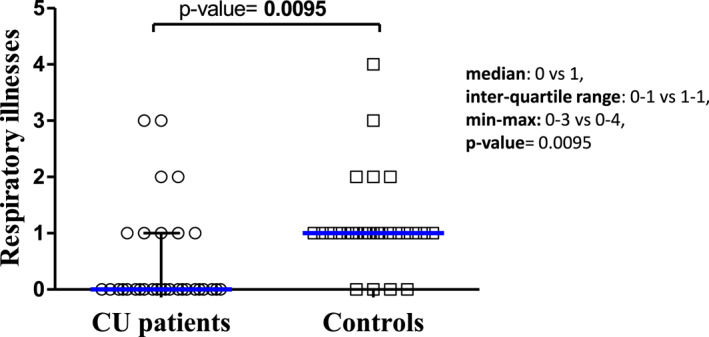
Scatter plot showing episodes suggestive of a viral infection of the upper respiratory tract in healthy controls and in patients with chronic spontaneous urticaria under omalizumab.

The CSU patients receiving omalizumab had significantly fewer episodes suggestive of URT infection than their paired healthy controls (median: 0 vs. 1, inter‐quartile range 0–1 vs. 1–1, min‐max: 0–3 vs. 0–4, respectively; *p* = 0.0095) (Figure 2). There was no difference concerning the duration (median 3 days, IQR 1–4 for both patients and controls).

## 4 DISCUSSION

In our study, omalizumab significantly reduced the number of common cold episodes in non‐atopic, non‐allergic CSU patients compared with healthy controls without affecting the illness duration. To the best of our knowledge, there are no other studies evaluating the role of omalizumab on respiratory illnesses in CSU patients, particularly those without known allergies or any atopic diathesis.

Our cohort consisted of CSU patients who responded to omalizumab. Therefore, high total IgE levels were expected since this subgroup of CSU patients has been shown to exhibit elevated total IgE,^20^, ^21^, ^22^ an observation that is especially prominent among non‐atopic CSU patients.^23^

In individuals with respiratory allergies, the overexpression of the high‐affinity IgE receptor, FcεRI, and their cross‐linking by the culprit allergens has been shown to disrupt virus‐induced IFN‐α responses,^10^, ^24^ providing an explanation of why patients with allergic asthma may experience more severe respiratory virus infections. Omalizumab reduces the expression of FcεRI receptors on the cell surface of basophils,^25^, ^26^ mast cells,^5^ and plasmacytoid dendritic cells (pDC)^27^, ^28^ by blocking the binding of allergen‐specific IgE to them and preventing receptor cross‐linking by the culprit allergens. Additionally, omalizumab increases virus‐induced IFN‐α responses, at least from pDC, in the presence of IgE cross‐linking. This enhanced interferon production limits cell‐to‐cell contamination, minimizing viral load and thus improving the organism's overall antiviral activity.^8^

Omalizumab has been shown to reduce viral respiratory illnesses, including rhinovirus (RV) infection, and the peak and duration of viral shedding in allergic asthmatic children and adolescents.^15^, ^16^ Treating these children with omalizumab seems to restore PBMCs' IFN‐α responses to rhinovirus or influenza. These improved responses have been associated with a lower risk of asthma exacerbations.^16^ Although the association between CSU and infections has shown indirectly^12^ or directly,^13^, ^14^ it is unknown if the aforementioned properties of omalizumab may apply to CSU pathophysiology and can explain the CSU control that omalizumab offers to these patients. If this is the case, then omalizumab administration may be considered not only in uncontrolled CSU patients after administration of regular sgAH doses but also in patients with controlled CSU experiencing frequent urticaria exacerbations due to respiratory illnesses.

A positive correlation between the expression of FcεRI receptors in inflammatory cells and serum total IgE levels has been shown in asthma, atopic dermatitis,^29^ and CSU.^30^ However, there is no evidence suggesting that non‐atopic CSU patients (that are expected to express more FcεRI receptors on their basophils) are more prone to respiratory infections. Furthermore, CSU is not considered, by definition, an atopic disorder. Two hypotheses can be formulated based on the evidence from the omalizumab studies on allergic asthma and the beneficial effect shown by the current study on non‐atopic CSU patients. The first hypothesis is that CSU occurs on top of or because of an unknown atopic background and that its susceptibility to infections is restored by omalizumab (similar to allergic asthma). The second hypothesis is that the normal immune responses to common respiratory infections that characterize the healthy population are further enhanced to a “super‐defense” status using omalizumab. To approach these hypotheses, firstly, the incidence of respiratory infections in CSU patients eligible for omalizumab but before starting omalizumab administration has to be recorded, and secondly, the effect omalizumab has on respiratory infection susceptibility in healthy individuals has to be studied.

Our study has a few limitations. The studied sample size was small, nasal wash samples were not obtained to detect the causative virus, and there was no placebo‐controlled group. There were also no immunological investigations to determine the potential antiviral mechanism of omalizumab in these patients. All these issues constitute unmet needs in these patients that need further evaluation. However, the pragmatic design of this study can more efficiently approach the effectiveness of such an intervention in real‐life, routine practice conditions for disorders such as common respiratory illnesses. Any placebo intervention to the negative controls would have been expected to increase the probability of a placebo response resulting from administering the inactive treatment, i.e., fewer respiratory infections. On the other hand, although this study had an interventional design, it was performed in chronic patients already under chronic treatment with omalizumab, thus minimizing the impact of any contextual effects in addition to any true intervention effect.^31^ Lastly, because of the nature of the disease and the need to typically follow the CSU treatment every 4 weeks for the administration of omalizumab (the study lasted until August 2016, while the European Medicines Agency approved omalizumab self‐administration in 2018), none of the patients dropped out of the study.

## 5 CONCLUSION

Omalizumab can reduce the number of common cold episodes in CSU patients and consequently may minimize viral‐related CSU exacerbations. This beneficial effect is exerted independently of the atopic status, even in non‐asthmatic individuals or non‐allergic patients without any evidence of respiratory susceptibility. Further large‐scale studies are needed to validate the current findings and elucidate the underlying relevant pathophysiology.

## AUTHOR CONTRIBUTIONS

George N. Konstantinou conceptualized the idea, performed the research, contributed to the statistical analysis, reviewed the article drafts, and did the final editing; Dimitrios Karapiperis contributed to the study and reviewed the article drafts; Indrashis Podde assembled the data, contributed to the statistical analysis and wrote the first draft. All authors have read the final version and agreed to it.

## CONFLICT OF INTEREST STATEMENT

George N. Konstantinou recently was a speaker and/or advisor for and/or has received research funding from AstraZeneca, Chiesi, GSK, Menarini, Novartis, Pfizer, Sanofi, and Vianex.

## Data Availability

Detailed data are available from the authors upon reasonable request.
